# Promoted Anodizing Reaction and Enhanced Coating Performance of Al–11Si Alloy: The Role of an Equal-Channel-Angular-Pressed Substrate

**DOI:** 10.3390/ma12193255

**Published:** 2019-10-05

**Authors:** Zhikai Zhou, Dan Song, Ningning Liang, Haiyang Jiang, Bo Gao, Yuna Wu, Aibin Ma, Yanxin Qiao, Jiapeng Sun, Jinghua Jiang, Xiaolong Ma

**Affiliations:** 1 College of Mechanics and Materials, Hohai University, Nanjing 210098, China; 18260056968@163.com (Z.Z.); wuyuna@hhu.edu.cn (Y.W.); aibin-ma@hhu.edu.cn (A.M.); sun.jiap@gmail.com (J.S.); 2 Suqian Research Institute of Hohai University, Suqian 223800, China; 3 School of Material Science and Engineering, Nanjing University of Science and Technology, Nanjing 210094, China; ningning623@126.com (N.L.); gaobo@njust.edu.cn (B.G.); 4 School of Metallurgy and Materials Engineering, Jiangsu University of Science and Technology, Zhenjiang 215600, China; justjhy@126.com (H.J.); yxqiao@just.edu.cn (Y.Q.); 5 Department of Materials Science and Engineering, Texas A&M University, College Station, TX 77845, USA

**Keywords:** anodizing coating, ECAPed substrate, microstructure, corrosion resistance, Al–Si alloy

## Abstract

In this paper, the effect of the equal-channel-angular-pressed (ECAPed) substrate on the coating formation and anticorrosion performance of the anodized Al–11Si alloy was systematically investigated. The ECAP process dramatically refines both Al and Si phases of the alloy. The parallel anodizing circuit is designed to enable a comparative study of anodizing process between the cast and the ECAPed alloys by tracking their respective anodizing current quota. The optimum coatings of both alloys were obtained after anodization for 30 min. The ECAPed alloy attained a thicker, more compact, and more uniform coating. Energetic crystal defects in the fine Al grains of the ECAPed substrate promote the anodizing reaction and lead to the thicker coating. Fragmented and uniformly distributed fine Si particles in the ECAPed alloy effectively suppress the coating cracks, enhancing the compactness of the coating. Overall, the ECAP-coated sample exhibits the best anticorrosion performance, which is evidenced by the concurrently enhanced prevention of coating and improved corrosion resistance of the substrate.

## 1. Introduction

Aluminum (Al) and its alloys are widely used lightweight structural materials, which have low density, high specific strength, excellent thermal conductivity, and great availability [1]. Generally, Al alloys have good corrosion resistance in less aggressive environments, owing to the efficient protection of its amorphous passive oxide film [2,3]. However, in humid environments and acidic or alkaline solutions, they still suffer severe corrosion damage [4]. Several aggressive ions, especially Cl^−^, accelerate the corrosion damage of Al alloys by forming soluble salts such as AlCl_3_ on the surface of metals, replacing the original hydroxide thin films. The severity of the corrosion damage is primarily determined by the competition between chloride ions and the hydroxide ions to bond with the Al ions. In other words, the integrity and stability of the hydroxide passive films in solutions are vital to the corrosion resistance of Al alloys [5]. An effective approach to increase the corrosion resistance of Al alloys is to fabricate coating layers on the surface to create barriers against direct corrosive attacks. Commercial anticorrosion coatings for Al alloys include electroplating [6], chemical conversion coating [7,8], and anodizing coatings [9,10]. Among them, anodizing coatings have the most widespread industrial application because of their ease of processing and low cost [11,12,13,14,15]. The quality of an anodizing coating, including thickness, uniformity, and compactness, is greatly influenced by both the anodizing parameters and the microstructure of the substrate alloy [16].

Another critical concern for application of Al alloys is their mechanical properties, including strength and ductility. In fact, achieving both superior mechanical properties and excellent corrosion resistance is a necessity for a long service life for Al alloys in an aggressive environment. Severe plastic deformation (SPD) techniques have been extensively reported to effectively enhance the mechanical properties of metallic materials [17,18,19,20,21]. For instance, excellent tensile properties (including high yield or ultimate stress and elongation), as well as extremely high impact toughness, can be obtained in Al alloys using equal-channel angular pressing (ECAP), especially via the multipass ECAP process [22,23]. The primary reason for the remarkable improvement in mechanical properties by ECAP processing is the effective microstructure refinement induced by imposed severe strain, which offers significant strengthening via grain boundary strengthening [24]. Despite the significant enhancement in mechanical properties, ECAP processing itself offers a limited improvement in the corrosion resistance of Al alloys [25,26,27,28], which is not acceptable for long service. Therefore, to meet the application requirements, additional processing to improve corrosion resistance is necessary for ECAPed Al alloys. Using an anodizing coating is a promising method to achieve this purpose. In addition, investigating the effect of ECAPed substrates on the anodizing process could also provide insight into understanding how the substrate microstructure influences the coating process, which is of both scientific and practical significance, but is yet not well-explored.

In this work, we will use an Al–Si alloy for study. The Al–Si series is the most important system of the Al alloys, because of its excellent castability, weldability, and superior wear resistance [29,30,31,32,33,34,35]. The content, size, scale, and distribution of the Si phase have been commonly believed to strongly affect the mechanical properties and castability of the alloys [36,37,38,39,40,41,42,43]. Al–Si alloys also have decent corrosion resistance in less aggressive environments. However, due to the potential difference between noble Si (standard electrode potential is −0.26 V_NHE_) and active Al (−0.85 V_NHE_), micro corrosion cells can be created, leading to weakened corrosion resistance and localized corrosion damage of the Al–Si alloys [44]. Thus, the anticorrosion performance of an Al–Si alloy is also highly influenced by the Si phase [45]. Current studies on anodizing coating on conventional Al–Si alloys also reveal that the Si phase remains inert and embedded in the coating during the reaction. Its appearance will dramatically deteriorate the compactness and the uniformity of the anodizing coating [46,47]. ECAP processing is expected to modify the size and distribution of the Si phase profoundly. However, its effect on the subsequent anodizing process is not yet well-understood.

In the present study, the ECAPed Al–Si alloy is further processed by anodizing coating process. The microstructure and corrosion resistance of both the substrates and the coatings are investigated. The ECAPed alloy with the optimized anodizing coating presented the best anticorrosion performance compared to all other samples, either with or without coatings. Meanwhile, the effect of the ECAPed substrate on the anodizing coating process; the formation of thicker, more compact, and more uniform coating; and the enhanced anticorrosion performance are systematically revealed and discussed.

## 2. Experimental

### 2.1. Processing Route

**Figure 1:**
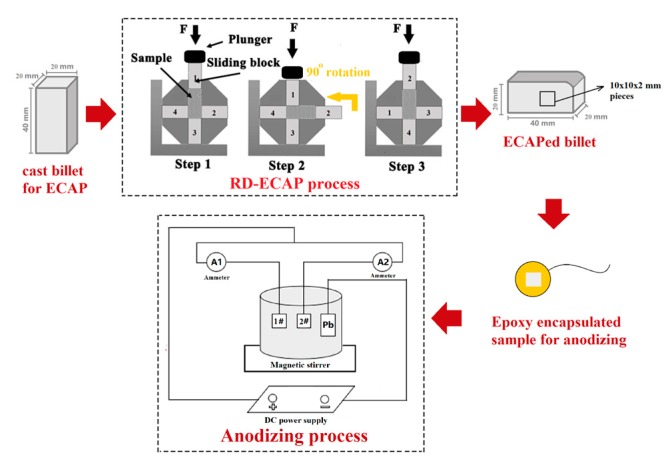
Schematic illustration of the combined process with rotary-die equal-channel angular pressing (RD-ECAP) and anodizing.

The raw material was cut from a commercial cast ingot of eutectic Al–11Si alloy (Aluminum Corporation of China Limited, Beijing, China). The chemical composition of this alloy was examined by a photoelectric direct reading emission spectrometer (Spectro Max, Kleve, Germany), the result of which are listed in Table 1. The original raw material is named cast alloy. As illustrated in Figure 1, the cast Al–Si alloy was processed by a combined process, which included ECAP and post-anodizing. The cast billets, with a size of 20 mm × 20 mm × 40 mm, were processed by multipass ECAP to obtain microstructure refinement in the alloy. The billets were pressed continuously for 8 passes using the rotary-die ECAP (RD-ECAP) technique with a displacement rate of 0.5 mm∙s^−1^ at 648 K. Detailed operation principles of RD-ECAP can be found in our earlier work [21]. Herein, the 8-pass ECAPed alloy is named the ECAPed alloy.

The samples for post-anodizing were cut from the ECAPed billet along the extrusion direction by electric discharge machining. Samples were embedded in epoxy with a squared area of 1 cm^2^ (1 cm × 1 cm square shape), exposed to a 20 wt.% sulfuric acid electrolyte at room temperature. The back of the sample was connected to copper wire for conduction. Before anodizing, the samples were mechanically polished, cleaned by acetone, and dried by air. During anodizing, one cast sample and one ECAPed sample were anodized concurrently within a parallel circuit (the bottom of Figure 1). A lead plate was selected as the cathode, and electromagnetic stirring was used to ensure the uniformity of the electrolyte. Benefiting from this circuit design, both samples were subjected to an identical voltage and exposed to the same chemical environment during the anodization. Thus, the external anodization parameters were precluded and only the differences in the internal microstructures of the substrates accounted for the resultant anodizing layer characteristics. The anodizing process was executed via a constant total input current of 0.06 A, and a nominal total anodizing current density of 0.03 A∙cm^−2^. Two ammeters were used to monitor the respective anodizing currents of each sample. After the anodizing coating, the samples were cleaned with deionized water and dried with hot air. Herein, the ECAPed alloy that has gone through the anodizing process is named the ECAP-coated sample, while the cast alloy that has gone through the anodizing process is named the cast-coated sample. To investigate the details of the evolution of microstructure characteristics, the cast and ECAPed alloys were processed using different anodizing durations of 10, 20, 30, and 40 min.

### 2.2. Microstructure Characterizations

The microstructures of the cast and ECAPed Al–Si substrate alloys, as well as the coated samples, were observed by scanning electron microscope (SEM; FEI 3D, Hillsboro, OR, USA). The samples for SEM observation were mechanically polished and etched using 1% hydrofluoric acid aqueous solution. The θ-2θ X-ray diffraction (XRD), ranging from 10° to 90°, was performed using a Bruker D8 Advance diffractometer (Bruker AXS, Karlsruhe, Germany) with Cu Kα1 radiation and a scan rate of 2 degrees/min. The microstructure of the ECAPed alloy was further characterized by transmission electronic microscope (TEM; FEI Tecnai G2 F20). The TEM sample was mechanically polished to about 40 μm, and further thinned and perforated by ion milling (Gatan 695C, Pleasanton, CA, USA). Electron backscattered diffraction (EBSD) characterization of the cast and ECAPed alloys was conducted via a SEM (Hitachi S-3400N, Tokyo, Japan) equipped with an oxford-instruments HKL-EBSD system (Oxford Symmetry, London, UK).

### 2.3. Corrosion Tests

The corrosion behavior of the substrate and coated samples in the 3.5 wt.% aqueous solution was investigated by electrochemical test and salt spraying corrosion test. A Parstat 2273 advanced potentiostat (Princeton, Oak Ridge, TN, USA) was used in the electrochemical tests. Both the substrate and coated samples in the electrochemical test had a squared exposure of 1 cm^2^. Note that the substrate samples were mechanically polished before tests, while the coated samples were tested without polishing. Electrochemical impedance spectroscopy (EIS) and potentiodynamic polarization (PDP) were employed in the electrochemical testing. EIS tests were conducted at frequencies ranging from 10 mHz to 10 kHz. The amplitude of the sinusoidal potential was 5 mV. The potential for PDP scanning ranged from 250 mV below the OCP to 250 mV above the OCP, at a scan rate of 1 mV∙s^−1^. Before EIS and PDP tests, the samples were pre-immersed in the solution for 2 h to reach the stable open circuit potential (OCP). To ensure data reproducibility, 5 parallel samples were tested with EIS and PDP testing, respectively.

The neutral salt spray test was conducted using the 3.5 wt.% NaCl solution at the salt spray ambient temperature of 35 °C. The optical macro and SEM micro corrosion morphologies of the samples were observed via an optical microscope (Hirox KH-7700, Hackensack, NJ, USA) and scanning electron microscope (FEI 3D, Hillsboro, OR, USA), respectively.

## 3. Results

### 3.1. Microstructure Characteristics of the Substrate and the Anodizing Coating

**Figure 2:**
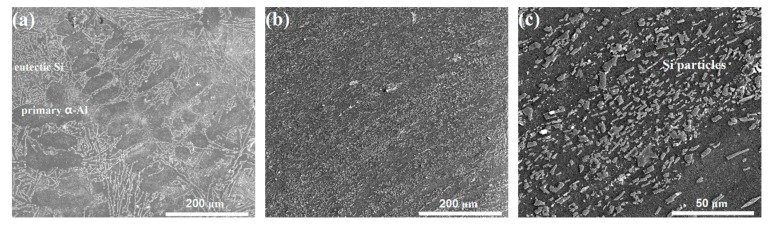
Scanning electron microscopy (SEM) observations of microstructures in the cast and the equal-channel-angular-pressed (ECAPed) Al–11Si alloy samples: (**a**) as-cast alloy; (**b**) ECAPed alloy with low magnification; (**c**) ECAPed alloy with high magnification.

Figure 2 shows the microstructures in the cast and ECAPed Al–11Si alloys. As shown in Figure 2a, the cast alloy possesses a typical eutectic microstructure, which includes the dendritic primary α-Al phase and eutectic structure (continuous, net-structured eutectic Si phase and Al phase). Figure 2b shows the microstructure of the ECAPed alloy along the extrusion direction. Prominently, the cast eutectic microstructure is entirely absent. Instead, due to the imposed severe strain, parallel strip features along the direction of plastic flow dominate the microstructure. The magnified micrograph in Figure 2c shows that the eutectic Si phase has been fragmented and refined into isolated fine Si particles. The microstructure evolution of the Al phase cannot be revealed by SEM observation.

**Figure 3:**
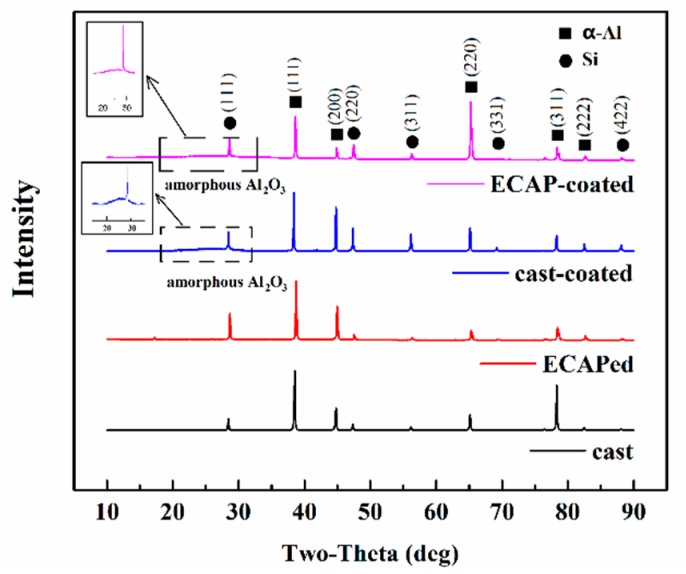
X-ray diffraction (XRD) results for the coated and uncoated samples.

XRD patterns of the substrate samples and the coated samples (30-min anodized samples) are presented in Figure 3. In the substrate sample, only the Al phase (JCPDS 04-0787) and Si phase (JCPDS 27-1402) were detected, as labeled in the pattern. No other intermetallic compounds were detected, probably because of the limited content of other alloying elements, such as Cu, Fe, Mn, Mg, and Zn. As expected, the Al phase and Si phase were also detected in both the cast-coated and the ECAP-coated samples. More importantly, broad peaks representing the amorphous alumina appear between 20° and 38° in both coated samples, as shown in the inset in Figure 3. Early studies [48,49] reported that the anodizing reaction products of Al alloys in acidic electrolytes were mainly composed of the amorphous alumina, which is consistent with our observation here.

**Figure 4:**
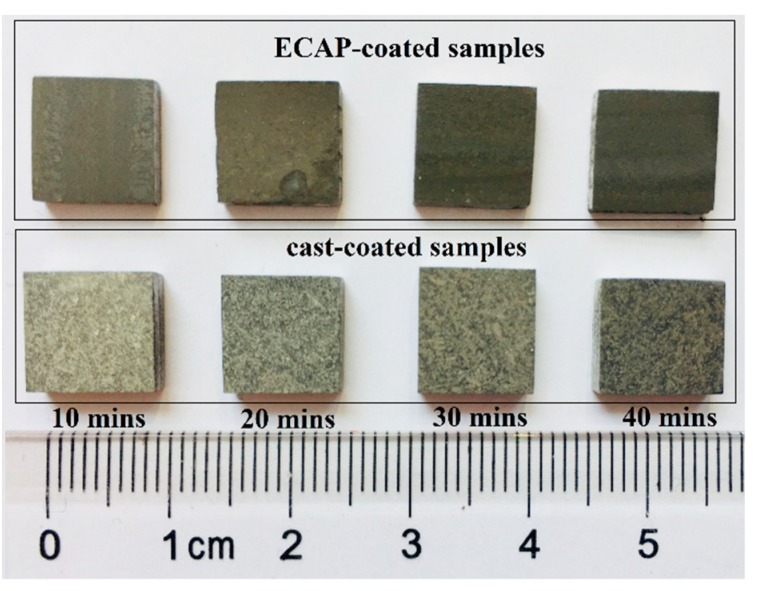
Optical macro morphologies of the coated samples anodized with different anodizing durations.

Figure 4 shows the optical macro surface morphologies of the anodizing coatings fabricated with different anodizing durations. Macroscopic compact coatings have been formed on all of the cast and ECAPed Al–Si alloy. The coatings show a remarkable gray color, which becomes darker for both coated samples as the anodizing duration increases. By comparison, one can easily see that the surfaces of ECAP-coated samples are darker than those for cast-coated samples anodized for the same duration. In addition, the cast-coated samples exhibit color variation on the surface, indicating the formation of a non-uniform coating microstructure caused by the composite nature of the substrate microstructure, as described earlier [47]. Conversely, the coatings of the ECAP-coated samples are more uniformly grey.

**Figure 5:**
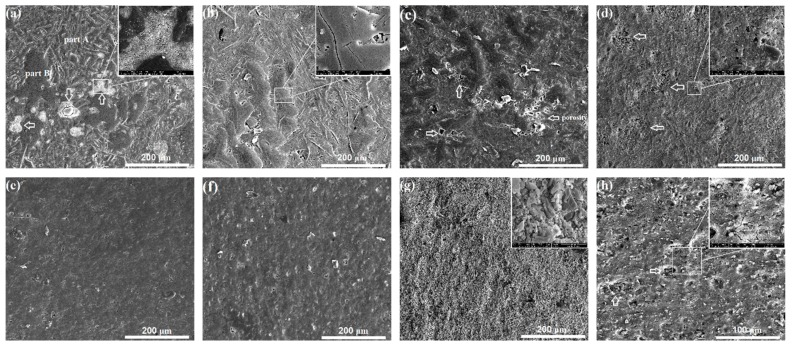
SEM surface morphologies of the coated samples with different anodizing durations: (**a**–**d**) the cast-coated samples anodized for 10, 20, 30, and 40 min, respectively; (**e**–**h**) the ECAP-coated samples anodized via 10, 20, 30, and 40 min, respectively.

Figure 5 shows the SEM surface morphologies of the coatings, with anodizing durations ranging from 10 to 40 min. As seen in Figure 5a, the coating on the cast alloy surface is not uniform after anodization for 10 min. Pores and cracks can be seen in the uncoated area, as marked by arrows and in the inset at the top right. In the coated region, two typical morphologies are present, labeled as part A and part B in Figure 5a. Part A exhibits typical strip patterns that are roughly equally spaced from each other. Part B shows a more uniform feature embossed on the sample. The distinctive characteristics in parts A and B are associated with the non-uniformity of the eutectic microstructure in the cast sample. Part A is likely the coating formed on the eutectic structure, while part B is the coating formed on the dendritic primary α-Al phase. In contrast, the surface morphology of the anodizing coating on the ECAP-coated sample is markedly different, as seen in Figure 5e. The coating covers the sample surface at the same magnification, and it is rather uniform.

After 20 min anodization, the coating gradually reaches a better coverage on the cast-coated sample. However, the non-uniform characteristic of the coating morphology still remains, and even leads to large and long micro-cracks on the surface, as shown in the inset in Figure 5b. Notably, the sites of those micro-cracks are associated with the dendritic primary α-Al phase. As for the ECAP-coated sample (as seen in Figure 5f), the results are similar to those obtained after 10 min anodization coating; the sample is quite uniform and compact.

After 30 min anodization, pores in the coating on the cast sample continue to grow (marked by white arrows in Figure 5c) and the non-uniform nature of the coating is still reserved. Changes also take place in the ECAP-coated sample. The surface is still uniform overall, but it presents localized coarsening and granulation. The inset of Figure 5g demonstrates the remarkable stacks of the granulated coating product, showing a size of 2–8 μm.

After 40 min anodization, the coating on the cast sample undergoes significant changes, as shown in Figure 5d. The uniformity improves as the coating products coarsens. It becomes difficult to distinguish the coatings on the dendritic primary α-Al phase and the eutectic structure. However, porous structures (as marked by white arrows and magnified at the top-right inset) are still present. As for the ECAP-coated sample, the coating experiences an even more prominent coarsening process, as shown in Figure 5h. Micro-cracks, micro-dimples, and even pulverization are widely found on the coating.

**Figure 6:**
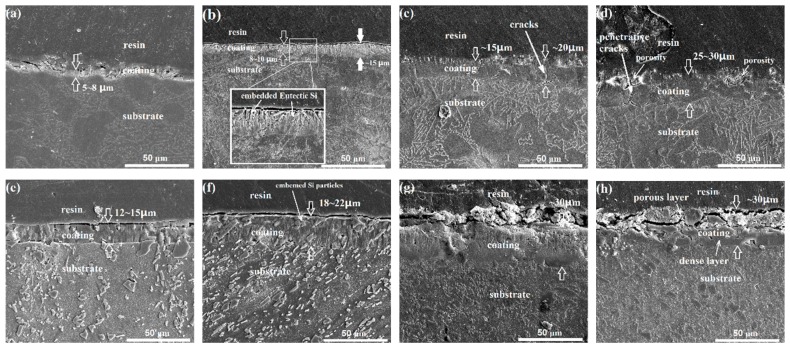
SEM cross-sectional morphologies of the coated samples with different anodizing times: (**a**–**d**) the cast-coated samples anodized for 10, 20, 30, and 40 min, respectively; (**e**–**h**) the ECAP-coated samples anodized for 10, 20, 30 and 40 min, respectively.

**Figure 7:**
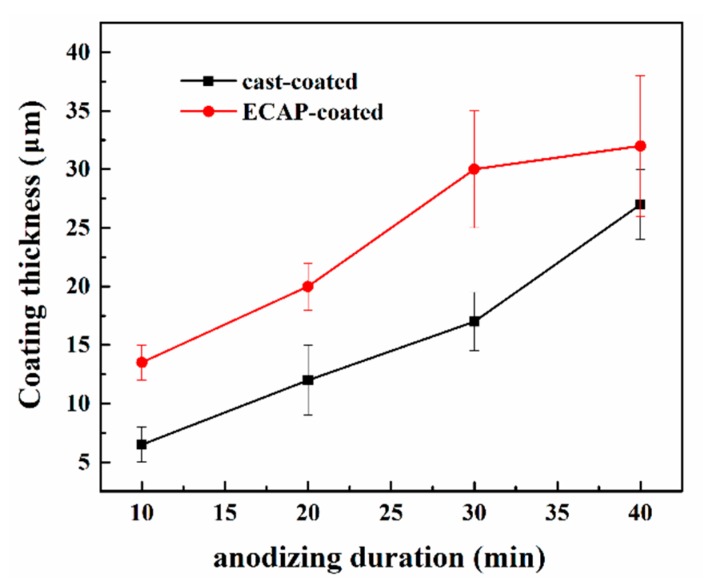
The coating thickness evolution during the anodizing reaction up to 40 min.

Figure 6 shows the SEM cross-sectional morphologies of the cast-coated and ECAP-coated samples anodized for 10, 20, 30, and 40 min. Figure 7 summarizes the measured coating thickness evolutions in both samples. Note that each data point is based on 15 SEM micrographs from 3 parallel samples. Notably, the ECAP-coated sample always has a thicker coating than the cast-coated sample with the same anodizing duration, indicating its overall faster coating process. This is even more prominent with anodizing durations of less than 20 min, where the coating thickness in the ECAP-coated sample is more than twice that in the cast-coated counterpart. In addition, the thickening process in the cast-coated sample is always accompanied by the concurrent development of pores and cracks (see Figure 6c,d), making it inferior in terms of resisting corrosion. As for the coating process of the ECAPed sample itself, its thickness reaches its maximum at 30 min. Afterward, the coating thickness does not change much, although its compactness and integrity degrade as the cracking and delamination start to appear (as marked on the 40-min-anodized sample in Figure 6h), which is consistent with the top view. Therefore, it can be concluded that the 30-min-anodized ECAP-coated sample was superior to all other samples because of its maximum coating thickness and better compactness.

### 3.2. Corrosion Behavior in NaCl Medium

**Figure 8:**
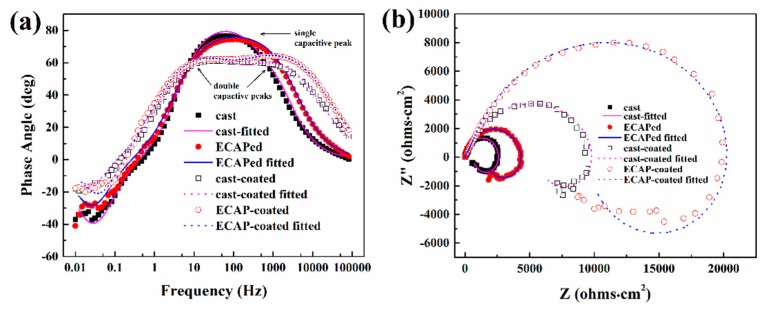
Electrochemical impedance spectroscopy (EIS) Bode plots (**a**) and Nyquist plots (**b**) of the coated and uncoated samples tested after immersion corrosion in 3.5 wt.% NaCl solution.

As the 30-min-anodized samples had the best overall coating quality (thickness, uniformity, and compactness) in both series, subsequent corrosion tests were performed on the 30-min-anodized cast-coated and ECAP-coated samples for further evaluations. Figure 8 illustrates the EIS Bode plots (Figure 8a) and Nyquist plots (Figure 8b) after immersion corrosion for 2 h. Coated samples show three time characteristics in the Bode plots, including the two capacitive arcs in the high and middle frequencies, and one inductive arc at low frequency. Double peaks (marked by black arrows) are the typical EIS characteristics for many coated samples, and they are related to the responses from the substrate and coating, respectively [50]. This is also the case in our study. Meanwhile, the uncoated substrate alloys present a single capacitive peak due to the single response from the substrate. Another minor note in the Bode plot is that the measured highest phase angles (HPA) are greater in the uncoated samples, which is likely caused by the much rougher surfaces on the coated samples. This is because the substrate alloys were mechanically polished before testing, while the top coating layers have to be retained on the coated samples for subsequent tests.

As for the interpretation of the Nyquist plots, the capacitive arc diameter is a reasonable indicator of the corrosion resistance. The larger the diameter is, the better corrosion resistance the experimental sample has [51]. In this study, both coated samples have much larger capacitive arc diameters than the substrate alloys. Meanwhile, the ECAPed and ECAP-coated samples have larger capacitive arc diameters than the cast and cast-coated samples, respectively. The *R*_s_(*C*_f_(*R*_pore_(*C*_dl_*R*_t_(*R*_L_L)))) and *R*_s_(*C*_dl_*R*_t_(*R*_L_L)) equivalent circuits are used to fit and further interpret the EIS data of the coated and uncoated samples, respectively. The fitted EIS parameters are listed in Table 2. In both equivalent circuits, *R*_s_ is the electrolyte solution resistance, *C*_dl_ and *R*_t_ represent the double-layer capacitance and the charge transfer resistance of the substrate–coating interface, and *R*_L_ and *L* represent the inductive loop. In addition, in the equivalent circuit used for the coated sample, *R*_pore_ and *C*_f_ represent the microporous resistance and capacitance of the coating–medium interface [52,53,54]. In the substrate–coating–medium corrosion system of our study, *C*_f_ reflects the compactness and integrity of the coating, and *R*_pore_ can be used to evaluate the corrosion resistance of the coating. Meanwhile, *C*_dl_ reflects the surface integrity of the substrate, and *R*_t_ can be used to evaluate the corrosion resistance of the substrate. As seen in Table 2, lower *C*_f_ values and greater *R*_pore_ values indicate better compactness and integrity, as well as better corrosion resistance, for the coatings of the ECAP-coated sample than that of the cast-coated sample. Furthermore, the ECAPed and ECAP-coated samples also have greater *R*_t_ values and lower *C*_dl_ values compared to the cast and cast-coated samples, which indicates better corrosion resistance and less corrosion damage for the ECAPed alloy and the substrate of the ECAP-coated sample, respectively. It was also reported by Curioni et al. [55] that *R*_L_ can be linked to the anodic reaction of corrosion. Herein, one may infer that the larger *R*_L_ values of the ECAP-coated sample can be related to its alleviated anodic reaction compared to the cast-coated sample. Given the above analysis, it can be concluded that the better corrosion resistance alleviated corrosion damage in both the coating and substrate of the ECAP-coated sample.

**Figure 9:**
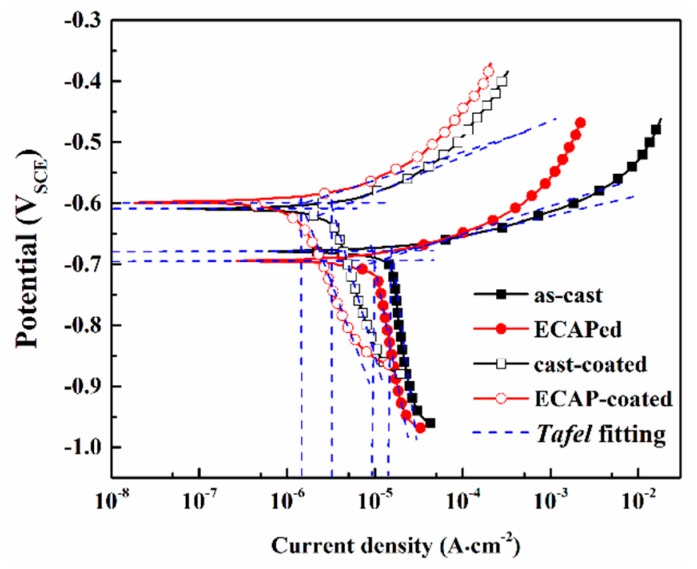
Potentiodynamic polarization curves for the coated and uncoated samples tested after immersion corrosion in 3.5 wt.% NaCl aqueous solution for 2 h.

PDP test results in Figure 9 demonstrate the corrosion behavior of the coated and uncoated samples under strong polarization. The corrosion potential (*E*_corr_), corrosion current density (*i*_corr_), anodic polarization rate (*β*_a_), cathodic polarization rate (*β*_c_), as well as polarization resistance (*R*_p_), are summarized in Table 3. Notably, the coated samples have much nobler *E*_corr_ values than that of the uncoated substrate alloy, and the ECAP-coated sample has the noblest *E*_corr_. This phenomenon should be ascribed to the formation of the anodizing coatings. Generally, the nobler values can present higher thermodynamic stability to corrosion for the tested sample. Corrosion current density reflects the corrosion kinetics, which usually evaluates the corrosion rate of the tested sample. As illustrated by the blue dash lines, *Tafel* extrapolation method was used to determine the current density. Compared to the cast alloy, the corrosion resistance of the ECAPed alloy is remarkably improved, presenting a lower *i*_corr_ value (decreased about 35%). The coated samples have a fifth *i*_corr_ in uncoated substrate alloys. In addition, the ECAP-coated sample has the lowest *i*_corr_ value among all samples, at only about 8.5% of that of the cast alloy. In addition to *i*_corr_, one can also find that the coated sample has a higher anodic polarization rate (*β*_a_) than that of the uncoated samples, and this phenomenon is more remarkable in the ECAP-coated sample. The higher anodic polarization rate indicates better protection of the coating against corrosion. Therefore, the coating of the ECAP-coated sample has better protective performance. Polarization resistance (*R*_p_) can also be used to evaluate the resistance of the corrosion system, which can be calculated via the following equation.

(1) $$R_{P}=\frac{\beta_{\alpha }\beta_{c}}{2.3\left(\beta_{\alpha }+\beta_{c}\right)i_{corr}}$$

Larger *R*_p_ values reflect better corrosion resistance. In the present study, the ECAPed alloy has a larger *R*_p_ value than the cast alloy. Meanwhile, the coated samples have larger *R*_p_ values than the uncoated substrate alloy, and the ECAP-coated sample has the largest one.

**Figure 10:**
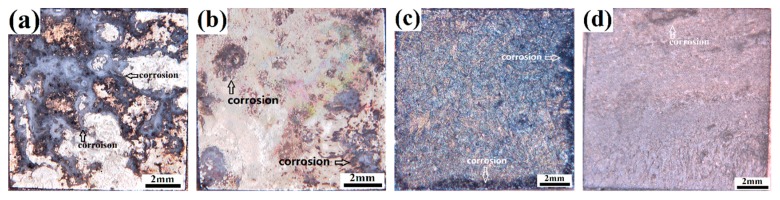
Optical corrosion morphologies of the samples after salt spray testing for one day: (**a**) cast alloy; (**b**) ECAPed alloy; (**c**) cast-coated sample; (**d**) ECAP-coated sample.

The corrosion behavior of the substrate alloys and the coated samples in the accelerated chlorine salt environment was studied via neutral salt spray testing. The corrosion damage and the corrosion resistance of the samples were evaluated in parallel by the optical macro and SEM micro corrosion morphologies. Figure 10 presents the optical corrosion morphologies of the samples after salt spray testing for one day. As marked, the continuous salt spray erosion leads to severe corrosion damage in the substrate alloys, while the damage in the coated samples is limited. Moreover, it can be found that the corroded area (black area) of the ECAPed alloy is less than that of the cast alloy, indicating less corrosion damage and better corrosion resistance. The macroscopic corrosion damage in the coated samples is not severe; only limited darker corrosion spots can be found at the edges of the samples.

**Figure 11:**
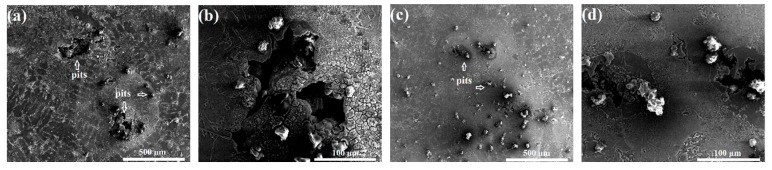
SEM corrosion morphologies of the cast and ECAPed alloys after salt sprayi testing for 1 h: (**a**,**b**) the cast-coated sample at low and high magnification, respectively; (**c**,**d**) the ECAP-coated sample at low and high magnification respectively.

Figure 11 shows the SEM corrosion morphologies of the cast and ECAPed alloys after salt spray testing for 1 h. It is worth emphasizing that the anodizing coatings of both coated samples still remain intact and no apparent corrosion trace can be found via SEM. Corrosion pits have been created on the substrate alloys due to the corrosion-induced dissolution of the Al matrix. Due to the electrode potential difference between the Al phase (anode with lower potential) and the Si phase (cathode with more noble potential), the Al phase is corroded under the concurrent action of chloride ion erosion and micro-galvanic corrosion. Relatively, the ECAPed alloy has a greater number of corrosion pits but smaller pit sizes, and thus suffers less corrosion damage. Another noteworthy observation is that the corrosion pits propagate into the matrix without leaving any coverage of corrosion products.

**Figure 12:**
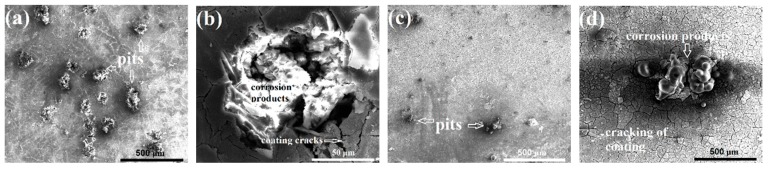
SEM corrosion morphologies of the coated samples after salt spray testing for one day: (**a**,**b**) the cast-coated sample at low and high magnification, respectively; (**c**,**d**) the ECAP-coated sample at low and high magnification, respectively.

Figure 12 shows the SEM corrosion morphologies of the coated samples after salt spray testing for one day. Note that those images were obtained from the non-severely corroded areas, which are away from corrosion spots marked by white arrows in Figure 10c,d. The coated samples suffered corrosion damage both in the coating and substrate, presenting typical localized pitting corrosion damage. The morphology of those pits was quite different from that of the uncoated sample. Magnified observations in Figure 12b,d show that the pits are covered by corrosion products, and there is limited corrosion propagating into the substrate. For the coated samples, the coating will provide sufficient prevention against Cl^−^ aggression. However, due to the imperfect compactness of the coating, the penetration of Cl^−^ through the coating into the substrate cannot be completely inhibited. The Cl^−^ attack will lead to the dissolution of the Al matrix and the formation of corrosion products inside. As a result, volume expansion will occur, further leading to the localized damage of the anodizing coating, including cracks in the coating around the pits. Judging from the pitting size and number, the corrosion damage of the ECAP-coated samples was significantly less than that of the cast-coated sample.

## 4. Discussion

From the increased *R*_t_ and *R*_pore_ values in EIS plots, the nobler *E*_corr_, higher *R*_p_, and lower *i*_corr_, as well as the visible alleviated corrosion damage shown in salt spray testing, it can be concluded that the ECAP-coated Al–Si alloy sample presents the optimum anticorrosion performance. The vast improvement in corrosion resistance is presumably caused by the concurrent improvement in both the substrate and the coating. In this session, the mechanisms of the better anodizing coating and anticorrosion performance of the ECAP-coated sample will be discussed.

### 4.1. Effect of Fine Al Grains in the ECAPed Substrate on the Promoted Anodizing Reaction

Generally, the electrochemical process of the anodizing reaction is strongly dependent on the electrolyte solution and the applied electrical parameters, such as anodizing voltage, current density, and the applied waveform. In addition, the internal factor (e.g., the substrate microstructure) also plays a critical role in the formation and performance of coatings. In the experimental set up of the present study, the parallel circuit design enables a same applied voltage for both samples, so that the effect of the substrate microstructure can be separated for further investigation by direct comparison of the measured currents through ECAPed and cast samples.

**Figure 13:**
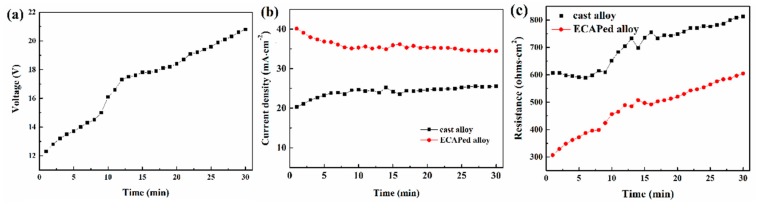
Evolution curves of (**a**) the applied anodizing voltage, (**b**) the individual anodizing current density, and (**c**) the individual anodizing reaction resistance.

Figure 13 shows the measured and calculated electrical parameters in the anodizing reaction system. As described earlier, the anodizing process was executed via the constant total input current (0.06 A). However, as the coating formation continues, the resistances of samples become higher and make the total voltage increase as well, as shown in Figure 13a. Individual measurement of current densities through two samples in Figure 13b shows that the current density is always higher in the ECAPed sample than in the cast alloy, indicating that the anodization is eternally faster in the ECAPed alloy. This seems inconsistent with thickness measurements in Figure 6 and Figure 7 at first glance, because at the end of the anodization, the measured thicknesses in both samples are comparable. Two possible issues account for this. First, we note the coating on the ECAPed sample after 30 min is loose enough that it can be easily peeled off during sample preparation. This is also the reason why the coating thickness of the ECAP-coated sample shows a similar value at 40 min compared to that at 30 min. Second, the increasing growth of thickness on the cast sample after 30 min is partially attributed to the rapid development of voids and pores, which take up a considerable volume but do not contribute any current. The drop and increase in the current densities of both samples during the initial period should be ascribed to the relative change of resistance in the samples. Figure 13c depicts the calculated anodizing reaction resistances versus the anodizing time. Notably, the anodizing reaction resistance of the cast alloy does not change much during the first 10 min. This is very likely because the coating layer is yet to fully cover the sample surface (i.e., the anodizing reaction is not affected too much). In contrast, the ECAPed sample has an increasing anodizing reaction resistance almost from the very beginning. This agrees well with the formation of the coating with better compactness and integrity (see Figure 6e). Later, the anodizing reaction resistances of both samples increase in a similar manner. Overall, it is straightforward to see that the anodizing reaction resistance is lower and the current density is higher in the ECAPed sample, indicating a higher coating efficiency.

**Figure 14:**
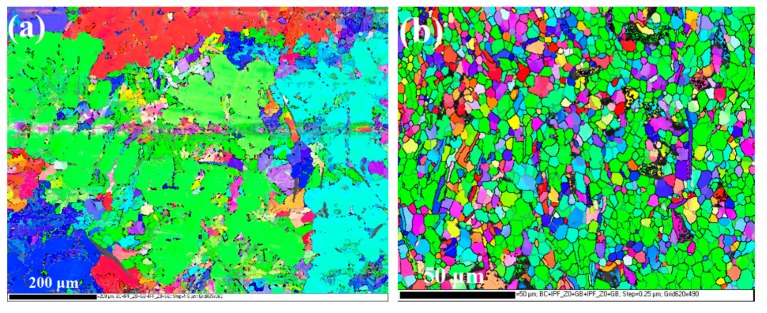
Electron backscattered diffraction (EBSD) inverse pole figure (IPF) mapping of the substrate alloys: (**a**) cast alloy; (**b**) ECAPed alloy.

**Figure 15:**
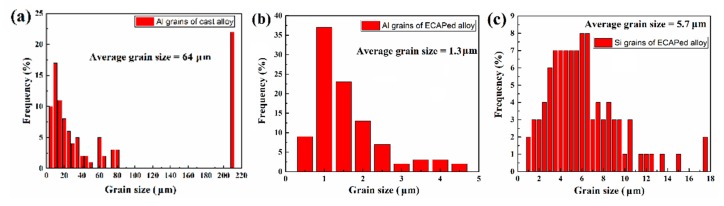
Grain size distribution of the Al and Si phases: (**a**) Al grains of cast alloy; (**b**) Al grains of ECAPed alloy; (**c**) Si grains of ECAPed alloy.

**Figure 16:**
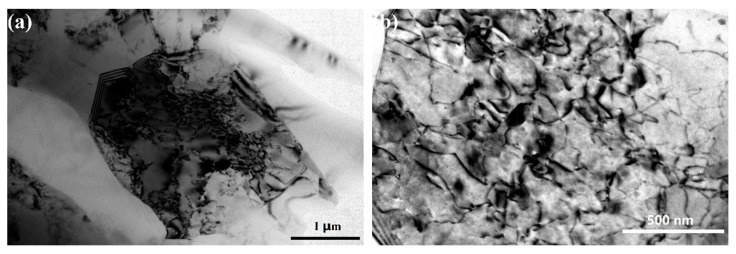
Transmission electronic microscopy (TEM) micrographs of Al grains in the ECAPed alloy: (**a**) grain size and morphology; (**b**) dislocation tangle in the grain interior.

To further understand the key microstructural characteristics that lead to the different coating formation processes, EBSD and TEM characterization were conducted. As shown in Figure 14 (EBSD inverse pole figure (IPF) mappings) and Figure 15 (grain size distributions), the average grain size of the Al phase in the cast alloy is about 64 μm. The coarse Al grains (grain size > 5 μm) have a number frequency of 90% in the cast alloy. The substrate microstructure of the ECAPed alloy is governed by the heavily refined Al grains and fragmented Si phase. The fine Al grains (1 μm < grain size < 5 μm) take up 100%, and the average grain size is about 1.3 μm. Ultrafine Al grains (grain size ≤ 1 μm) are also observed, which take up about 9% of the total number of scanned Al grains. The grain refinement and fragmentation of the Si phase are also remarkable, with the average size refined down to 5.7 μm after ECAP (see Figure 15c). Smaller grain size amounts to higher grain boundary density. Grain boundaries are usually of higher energy and less stability than the grain interior. In addition, they also offer shortcuts for diffusion needed for the anodizing reaction. Hence, they can provide a higher driving force and faster kinetics to promote the coating nucleation and the thickening process under the same applied voltage during the anodizing reaction. Figure 16a shows a typical TEM micrograph of the Al phase in the ECAPed alloy, where a grain with a size of about 2 μm can be found. As reported in many ECAPed materials, the formation of such fine grains is caused by the continuous evolution of dislocations, from discrete dislocations to tangles, cells, walls, and eventually sub-grain boundaries or new grain boundaries, to achieve grain refinement caused by the sufficient imposition of strain [56,57,58]. It should be noted that due to the relatively high ECAP executive temperature (648 K), the dynamic recovery and recrystallization is presumably concurrent during the ECAP process. However, in our case, there are still abundant tangled dislocations that remain inside the grains, as seen in Figure 16b. Similar to grain boundaries, dislocations can also promote the anodizing reaction from both thermodynamics and kinetics perspectives.

The fine grains with profuse dislocations in the ECAPed substrate significantly change the anodizing coating process from both thermodynamics and kinetics perspectives. First, the stored energies, including grain boundary energy and elastic dislocation energy, offers a much higher driving force to the initial coating nucleation on the ECAPed sample. Meanwhile, in kinetics, grain boundaries and dislocations act as shortcuts for atom diffusions for the coating formation. This is the dominant reason explaining the initially higher current density and the lower resistance in the ECAPed sample, as shown in Figure 13. As the anodizing reaction continues, the faster formation of the coating layer builds up a new barrier for diffusion and the anodizing reaction gradually transitions from nucleation control to diffusion control. In other words, the newly formed coatings offset the thermodynamic advantage in the ECAPed sample to a certain extent. Therefore, an immediate rapid drop and an increase of current density are seen in the ECAPed and the cast sample, respectively, given a fixed total current input in the circuit. When the anodizing reaction continues to proceed, diffusion starts to govern the reaction rate as the coating become thicker. Thus, the thermodynamic advantage no longer plays a decisive role in the reaction rate. However, the relatively more rapid diffusion through grain boundaries and dislocations still makes the anodizing reaction faster in the ECAPed alloy.

### 4.2. Effect of Fragmentation of the Eutectic Si Phase on Enhancing Coating Compactness

It should be noted that apart from the faster formation of the thicker coating, the presented advantages of the ECAP-coated sample over the cast-coated sample also include the better coating compactness and uniformity. This is largely determined by the fraction, morphology, and distribution the of eutectic Si phase [46,59]. In Figure 6b,e, long rod-like eutectic Si and discrete, small Si particles are commonly embedded in the anodizing coating of the cast-coated and ECAP-coated samples. This phenomenon is, in fact, present in all coated samples with various anodizing times. As the reaction proceeds from the top to the substrate interior, the Si phase is not involved in the anodizing reaction and remains intact after the reaction due to its insulating nature. However, the anodizing reaction product will change the volume of the original Al phase, and thus generate an internal strain at its interface with the Si phase. This strain relaxation leads to micro-cracks and micro-pores between the Si phase and the coating product. A similar phenomenon was also reported by Zhu et al. [59]. For the cast-coated sample, the eutectic Si phase is locally agglomerated in a network-like manner. It connects the micro-cracks or micro-pores and enables the formation of long and large cracks along the Si phase. Notably, such cracks can even evolve into penetrative ones from the top surface of the coating into the coating–substrate interface as the anodizing duration continues. For the ECAP-coated sample, the Si phase in the substrate has been fragmented into fine and discrete Si particles. Although micro-cracks and micro-pores are still possibly created around each individual Si particle in the coating, no destructive or penetrative cracks are present until 30 min of anodizing. As the anodizing continues to 40 min, the coating integrity of the ECAP-coated sample deteriorates as well, probably because the accumulation of the internal strain has reached its limit.

The Si phase also influences the coating uniformity as well. As seen in Figure 6b, the coating thickness varies a lot from spot to spot in the cast-coated sample. It is commonly found that the thicker coating develops in the dendritic primary α-Al phase, while the thinner coating is formed based on the eutectic structure. In the eutectic structure, consisting of both the Al phase and Si phase, the local availability of the Al content limits the anodizing reaction and results in a thinner coating. Moreover, the Si phase may partially inhibit the reaction when it intersects with the reaction front. For the coating of the ECAP-coated sample, the Al-rich dendritic phase and Al-poor eutectic structure are completely mixed, and the substrate presents a relatively uniform distribution of both the Al content and Si phase. As a consequence, the coating of the ECAP-coated sample presents a much more uniform thickness compared to that of the cast-coated sample.

**Figure 17:**
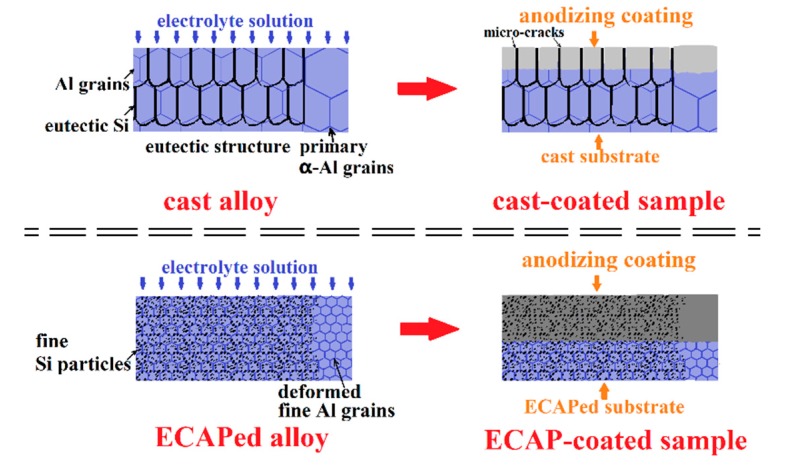
Schematic illustration of the anodizing coating process of the cast-coated and ECAP-coated samples.

To better understand the mechanism for the revealed improvement on coating thickness and compactness of the ECAP-coated sample, the schematics of the anodizing coating formation process and the corresponding microstructure alteration in all samples are presented in Figure 17 for comparison. Two major microstructural factors of the ECAPed substrate can be concluded to explain the better coating quality of the ECAP-coated sample. The thicker coating results from the intensified anodizing reaction promoted by the energetic microstructure characteristics, including grain boundary density and profuse dislocations, which are detailed in Section 4.1. The better coating compactness primarily results from the complete fragmentation of the eutectic Si structure, which depresses the formation of micro-cracks.

### 4.3. Mechanism of the Significantly Improved Anticorrosion Performance of the ECAP-Coated Sample

Generally, the anticorrosion performance of a coated sample depends mainly on two aspects, namely the corrosion resistance of both the coating and the substrate. In this study, the enhanced protection from the anodizing coating and the improved corrosion resistance of the substrate can be argued to elucidate the significantly improved anticorrosion performance of the ECAP-coated sample at a microscopic level.

The first factor is the enhanced protection from the thicker, more compact, and more uniform anodizing coating in the ECAP-coated sample. Coatings act as strong barriers against the penetration of the corrosive medium, especially during the initial corrosion stage. Therefore, the uncoated substrate alloys in Figure 10 were severely corroded in the salt spraying corrosion test, while both coated samples only suffered limited corrosion damage to the surfaces of the coatings. It is reasonable to conclude that a thicker coating can offer better resistance and prolong the protection against the penetration of the corrosive medium. The loss of coating compactness, especially the appearance of large-scale and penetrative cracks, deteriorates the protection significantly. Such cracks provide shortcuts for the corrosive medium into the coating–substrate interface and accelerate the corrosion process. Thus, the more compact coating in the ECAP-coated sample enables a substantially improved protection against corrosion.

The second factor is the improved corrosion resistance of the substrate. Once the corrosive medium travels to the coating–substrate interface through the coating, the corrosion reaction occurs on the substrate, leading to both the dissolution of the substrate and the localized damage of the coating. Consequently, penetration of the subsequent corrosive medium becomes even faster and easier, significantly accelerating the corrosion damage of the coated samples. As revealed in the electrochemical test and salt spray test, the ECAPed alloy presented remarkably improved corrosion resistance over the cast alloy. A similar improvement was also found in our previous studies on 32-pass ECAPed Al-11 wt.% Si alloy [26], 32-pass ECAPed hypereutectic Al-23% Si [25], and Al-26% Si [27]. The improvement in corrosion resistance of the ECAPed Al–Si alloy can be attributed to the following three factors. The first is the formation of the more stable oxide film in the air or the hydrated oxide film in the solution on the ECAPed alloy. Strain-induced grain refinement in Al grains offers more sites for nucleation and more shortcuts for diffusion to form denser and thicker oxide/hydroxide films, which are more resistant against the aggressive medium. The second is the significant decrease in micro-galvanic corrosion between the fine Si particles and Al grains. Fragmentation of the coarse eutectic Si phase into fine particles during ECAP weakens the cathodic effect of the Si phase, leading to the decreased corrosion in Al grains. The third is the more uniform corrosion instead of localized severe pitting corrosion. The uniform distribution of the fine Si particles significantly suppresses the localized corrosion tendency.

## 5. Conclusions

In summary, the significantly refined microstructure of the Al–11Si alloy was achieved via multi-pass ECAP process. The cast and ECAPed Al–11Si alloy were simultaneously anodized in the 20 wt.% sulfuric acid electrolyte via a parallel circuit under a constant total input current. The microstructure characteristics of the anodizing coatings, as well as their anticorrosion performance, were systematically studied. The main conclusions are:

- Optimum anodizing coating for both coated samples can be fabricated after anodizing reaction for 30 min. There is a competition between the cast and the ECAPed alloys in obtaining the quota of the anodizing current. During the whole anodizing duration, the anodizing current of the ECAP-coated alloy is always higher than that of the cast-coated alloy.
- The ECAP-coated sample has a thicker, more compact, and more uniform anodizing coating compared to the cast-coated sample. Fine Al grains with profuse grain boundaries and dislocations promote the anodizing reaction from both thermodynamics and kinetics perspectives, which leads to a thicker coating. The fragmentation of the eutectic Si into uniformly distributed fine Si-particles in the ECAPed alloy effectively suppresses the development of microscale porosity and cracks in the coating, leading to enhanced coating compactness and uniformity.
- The ECAP-coated sample has the optimum anticorrosion performance, presenting the lowest *i*_corr_ and largest *R*_p_ in PDP test, largest *R*_t_ and *R*_pore_ in EIS test, and the least corrosion damage in the salt spray test. The enhanced protection from the anodizing coating and the improved corrosion resistance of the substrate are the two factors contributing to the improved anticorrosion performance of the ECAP-coated sample.

## Figures and Tables

**Table 1:** Chemical composition of Al–11Si alloy (mass fraction, %).

Si	Cu	Fe	Mn	Mg	Zn	Al
11.10	0.95	0.86	0.22	0.24	0.32	Balance

**Table 2:** Fitted result of the EIS spectra.

Samples	*R_s_* (Ω∙cm^2^)	*C_f_* (μF∙cm^−2^)	*R*_pore_ (Ω∙cm^2^)	*C*_dl_ (μF∙cm^−2^)	*R*_t_ (Ω∙cm^2^)	*R*_L_ (Ω∙cm^2^)	*L* (H∙cm^2^)
Cast	9.5	/	/	18	646	2000	5355
ECAPed	8.3	/	/	15	1600	2814	10,200
Cast-coated	9.3	3.37	9376	14.2	1020	9206	89,180
ECAP-coated	8.9	3.55	19,450	7.2	1774	11,900	78,420

**Table 3:** Electrochemical parameters of the samples obtained via potentiodynamic polarization (PDP) tests.

Samples	*E*_corr_ (V)	*i*_corr_ (μA∙cm^−2^)	*β*_a_ (mV∙dec^−1^)	*β*_c_ (mV∙dec^−1^)	*R*_p_ (Ω∙cm^2^)
Cast	−0.69	15.2	32	1130	890
ECAPed	−0.67	10.1	43	820	1760
Cast-coated	−0.61	3.2	64	460	7630
ECAP-coated	−0.60	1.3	67	340	18,700
